# E-Learning Research Trends in Higher Education in Light of COVID-19: A Bibliometric Analysis

**DOI:** 10.3389/fpsyg.2021.762819

**Published:** 2022-03-03

**Authors:** Said Khalfa Mokhtar Brika, Khalil Chergui, Abdelmageed Algamdi, Adam Ahmed Musa, Rabia Zouaghi

**Affiliations:** ^1^University of Bisha, Bisha, Saudi Arabia; ^2^University of Oum El Bouaghi, Oum El Bouaghi, Algeria; ^3^Binghamton University, Binghamton, NY, United States; ^4^University of Rochester, Rochester, NY, United States

**Keywords:** e-learning, higher education, COVID-19, bibliometric analysis, Web of Science (WoS) database

## Abstract

This paper provides a broad bibliometric overview of the important conceptual advances that have been published during COVID-19 within “e-learning in higher education.” E-learning as a concept has been widely used in the academic and professional communities and has been approved as an educational approach during COVID-19. This article starts with a literature review of e-learning. Diverse subjects have appeared on the topic of e-learning, which is indicative of the dynamic and multidisciplinary nature of the field. These include analyses of the most influential authors, of models and networks for bibliometric analysis, and progress towards the current research within the most critical areas. A bibliometric review analyzes data of 602 studies published (2020–2021) in the Web of Science (WoS) database to fully understand this field. The data were examined using VOSviewer, CiteSpace, and KnowledgeMatrix Plus to extract networks and bibliometric indicators about keywords, authors, organizations, and countries. The study concluded with several results within higher education. Many converging words or sub-fields of e-learning in higher education included distance learning, distance learning, interactive learning, online learning, virtual learning, computer-based learning, digital learning, and blended learning (hybrid learning). This research is mainly focused on pedagogical techniques, particularly e-learning and collaborative learning, but these are not the only trends developing in this area. The sub-fields of artificial intelligence, machine learning, and deep learning constitute new research directions for e-learning in light of COVID-19 and are suggestive of new approaches for further analysis.

## Introduction

The idea of e-learning was originated in the 1990s to explain learning thoroughly through technical advances. When instructional architecture and technologies have advanced, more attention has been paid to studying with the pedagogy. University education, further education, and e-learning have also recently adopted prominent roles in e-learning, too. It is now possible to provide e-learning for off-the-formal training through the internet. It also increased the need for personalization and advanced social people’s tools (Siemens, 2005). In addition, it is often referred to as being able to read. It will help mix much learning more conveniently, but it has to be done, given the success of “traditional” e-learning pages. When the educational and technological assets join, this will be something more than a personal matter.

The COVID-19 pandemic has forced the closure of many activities, especially educational activities. To limit the spread of the pandemic, universities, institutes, and academic schools had to switch to e-learning using the available educational platforms. Social distancing is critical, and the COVID-19 pandemic has brought an end to face-to-face education, negatively impacting educational activities (Maatuk et al., 2021). This closure has stimulated the growth of distance education activities as an alternative to face-to-face education in their various forms. Accordingly, many universities have shared the best ways to deliver course materials remotely, engage students, and conduct assessments.

The concept of e-learning, although widely known has not yet been fully explored (Nicholson, 2007). Many countries designed and deployed distance education systems during the COVID-19 pandemic to ensure that higher education could continue without interruption (Tesar, 2020). Several opportunities and challenges related to e-learning, higher education, and COVID-19 arose as a result of this, prompting a flurry of research into the area. When looking at the scientific studies published during the COVID-19 pandemic, it shows clearly that many international journals have published a large number of academic articles about e-learning in higher education during COVID-19 (Karakose and Demirkol, 2021). Furthermore, a vast amount of bibliometric research has been carried out in this field. However, there is very little research focused entirely on the relationship between e-learning, higher education, and COVID-19, using scientometric or bibliometric analysis (Furstenau et al., 2021).

This paper will discuss bibliometric indicators for e-learning in higher education during COVID-19 studies and proceed with a network analysis to define the most important sub-areas in this topic. To define the trends of e-learning in higher education during COVID-19, the following questions are proposed:

*Q1: What are the most important sub-fields of e-learning in higher education in light of COVID-19?*

*Q2: Who are the most influential authors on the subject of e-learning in higher education in light of COVID-19?*

*Q3: What countries and research institutions are the most referenced for research on the subject of e-learning in higher education in light of COVID-19?*

*Q4: What are the research gaps and recent trends in the subject of e-learning in higher education in light of COVID-19?*

An analysis was conducted to provide a broad and long-term perspective on the vocabulary of learning publications. It helps to recognize emerging problems within the multifaceted and increasing study fields of the world of e-learning. Newly published studies can improve knowledge and bridge the knowledge gap through findings regarding e-learning trends; this applies particularly to higher education due to the importance of knowing the latest information about distance learning and its methods. For this reason, the research is valuable for analyzing the volume of publications that have been made on the subject matter and to solidify the knowledge base on what has been studied by different expert researchers in education. So this will create new progress and new proposals to improve education in the event of a future pandemic.

In recent years, there has been an increasing interest in research within areas related to e-learning: online learning, blended learning, technology acceptance model, smart learning, interactive learning environments, intelligent tutoring systems, digital learning were reported (Oprea, 2014; Castro-Schez et al., 2020; de Moura et al., 2020; Kao, 2020; Nylund and Lanz, 2020; Pal and Vanijja, 2020; Patricia, 2020; Şerban and Ioan, 2020).

A substantial quantity of literature has been written and published on the bibliometric analysis of e-learning. These studies mainly aim to identify the most critical areas (keywords) of e-learning. Networks such as that conducted by Chiang et al. (2010) showed that the significant research areas in e-learning are as follows: Education and Educational Research, Information Science and Library Science, and Computer.

### Science/Multidisciplinary Applications

Cheng et al. (2014) analyzed data from 324 articles published between 2000 and 2012 in academic journals and conference proceedings from 2000 to 2012 to determine the vital research areas (the results identify six research themes in the field e-learning). Tibaná-Herrera et al. (2018a) used VOSViewer to conduct a bibliometric analysis of SCOPUS and SCImago Journal & Country Rank to establish the “e-learning” thematic category of scientific publications, thereby contributing to the discipline’s consolidation, accessibility, and development by researchers.

Bai et al. (2020) have also pursued similar work in analyzing 7,214 articles published in 10 journals on the subject of e-learning from 1999 to 2018; this study offers valuable hints on the future direction of how e-learning may evolve. Fatima and Abu (2019) examined 9,826 records from the Web of Science (WoS) database between 1989 to 2018 to identify significant contributions to the area of e-learning. The findings of this study show that the United States and the United Kingdom have contributed more than half of the research in e-Learning. According to a recent survey by Mashroofa et al. (2020), the University of London is the most prolific institution globally. According to the WoS database, the institution has published 131 studies on e-learning; the bibliometric analysis of 6,934 results revealed that the publications received 59,784 citations.

Hung (2012) employed text mining and bibliometrics to examine 689 refereed journal articles and proceedings, comparing them to these research results. These works are divided into two domains, each of which has four groups. The study’s findings now offer evidence that e-learning methods vary across top countries and early adopter countries.

There have been multiple previous attempts to do a systematic review of e-learning publications (Lahti et al., 2014; Zare et al., 2016; Garcia et al., 2018; Rodrigues et al., 2019; Araka et al., 2020; Valverde-Berrocoso et al., 2020), these studies mainly aimed to identify research areas, the most used and most important methods, and tools in e-learning.

Many studies have examined the results of e-learning publications through meta-analysis (ŠUmak et al., 2011; Lahti et al., 2014; Mothibi, 2015; Cabero-Almenara et al., 2016; Yuwono and Sujono, 2018).

The study’s contribution is that no controlled studies have compared differences in networks, models, and software outputs to define the most critical research areas in e-learning and the most influenced authors, organizations, and countries.

The study makes an important contribution to the analysis of current models and networks of e-learning in higher education during the COVID-19 pandemic, aiming to define the most critical research areas in e-learning and the most influenced authors, organizations, and countries. In addition, it looks at the framework of e-learning and its future research trends in light of COVID-19. This has been done through numerous investigations (Tibaná-Herrera et al., 2018a,c; Hilmi and Mustapha, 2020; López-Belmonte et al., 2021).

## Materials and Methods

### Bibliometric Data

**FIGURE 1:**
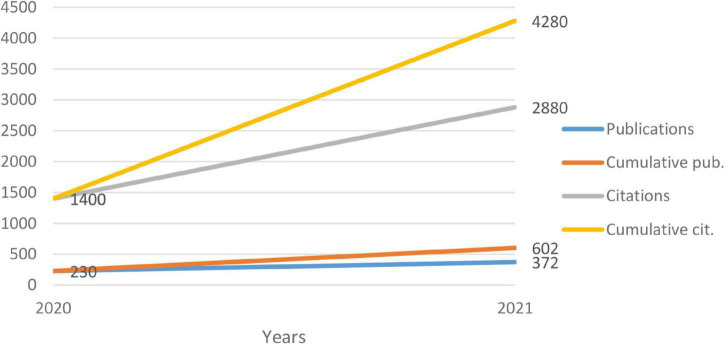
Publications per year (KnowledgeMatrix Plus outputs).

We retrieved published research *via* a topic search of the Science e-learning in higher education during the COVID-19 pandemic using the WoS database on August 12, 2021. The following search terms were used: topic = (“e-learning” “COVID-19” “higher education”), in title-abs-key from 2020 to 2021, and were 602 studies (475 articles, 80 articles; early access, 25 proceedings paper, 22 reviews) distributed over 2 years, as shown in Figure 1.

The following selection criteria were used to choose the studies. First, for the title, we looked at the following: the studies that looked at the topic of e-learning in higher education during COVID-19. Second, for the abstract, we looked at the following: the studies that addressed the problem of e-learning in COVID-19. Third, for the keyword, we looked at the following: the studies that included e-learning, higher education, universities, and COVID-19. Fourth, the subject areas were limited to a selection of works that dealt with this subject in the following disciplines: business management and accounting, educational sciences, social sciences, and psychology.

The bibliometric study data represents the overall research on “E-learning in higher education in light of the COVID-19” in the WoS database. These data covered the last 2 years (2020 and 2021) in which the use of e-learning was expected due to the closure and quarantine procedures.

The reasons for choosing this database over others, particularly Scopus and ScienceDirect, are due to several considerations; due to WoS data, the field of scientometrics has advanced significantly. WoS is more than simply a database of academic papers. Many information objectives are supported by this selected, organized, and balanced database, including full citation links and improved metadata (Birkle et al., 2020). WoS databases include high-quality research covering Science Citation Index Expanded (SCI-Expanded), Social Sciences Citation Index (SSCI), Arts & Humanities Citation Index (A&HCI), Emerging Sources Citation Index (ESCI) (Falagas et al., 2008).

Figure 1 illustrates how interest in e-learning research has increased in recent years, particularly between 2020 and 2021. Among the 602 studies with 4,280 citations, 230 in 2020 (1,400 citations), and 372 in 2021 (2,880 citations), the importance of higher education institutions, including universities, in this modern teaching and learning approach and their significance in the educational process during COVID-19 is evident. They are different from the periods approved in the previous studies (Chiang et al., 2010; Cheng et al., 2014; Bai et al., 2020; Fatimah et al., 2021). Therefore, this field of research (e-learning) has been renewed, and researchers should pay more attention to it to provide effective methods and approaches in light of the continuing epidemic.

### Methods and Tools

According to the methods and approaches of bibliometric analysis (see: Zupic and Čater, 2015, p. 04). the study relied on the co-occurrence indicator (co-word) to find out the main keywords on which previous studies focused as well as the co-authorship, publications, and citations indicators to find prominent authors, organizations, and countries in the topic of e-learning in higher education in COVID-19.

Following the methodology of preparing the bibliometric study in management and organization, which was explained by Zupic and Čater (2015), the bibliometric analysis was carried out by completing the following steps: research design, study questions, and analysis approach selection (co-occurrence, publication, citation, and co-authorship); bibliometric data compilation, selection, and filtration, analysis (choosing the appropriate bibliometric software, clean the data, and generate networks); visualization, and interpretation.

The bibliometric analysis was performed to design networks of e-learning and define the most frequent keywords and the most cited authors, organizations, and countries to explain new and current trends within this topic. This is achieved depending on different software: CiteSpace converts research domain concepts into mapping functions between research frontiers and intellectual bases and is effective for information visualization (Chen, 2016); VOSviewer is used to design the networks and is a powerful function for co-occurrence analysis and citation analysis (Van Eck and Waltman, 2013). KnowledgeMatrix Plus is a powerful tool for analyzing frequency and statistics (Chen and Song, 2017). This software was not used in previous studies (Chiang et al., 2010; Cheng et al., 2014; Bai et al., 2020; Fatimah et al., 2021).

## Results and Discussion

### Keywords Frequency

**FIGURE 2:**
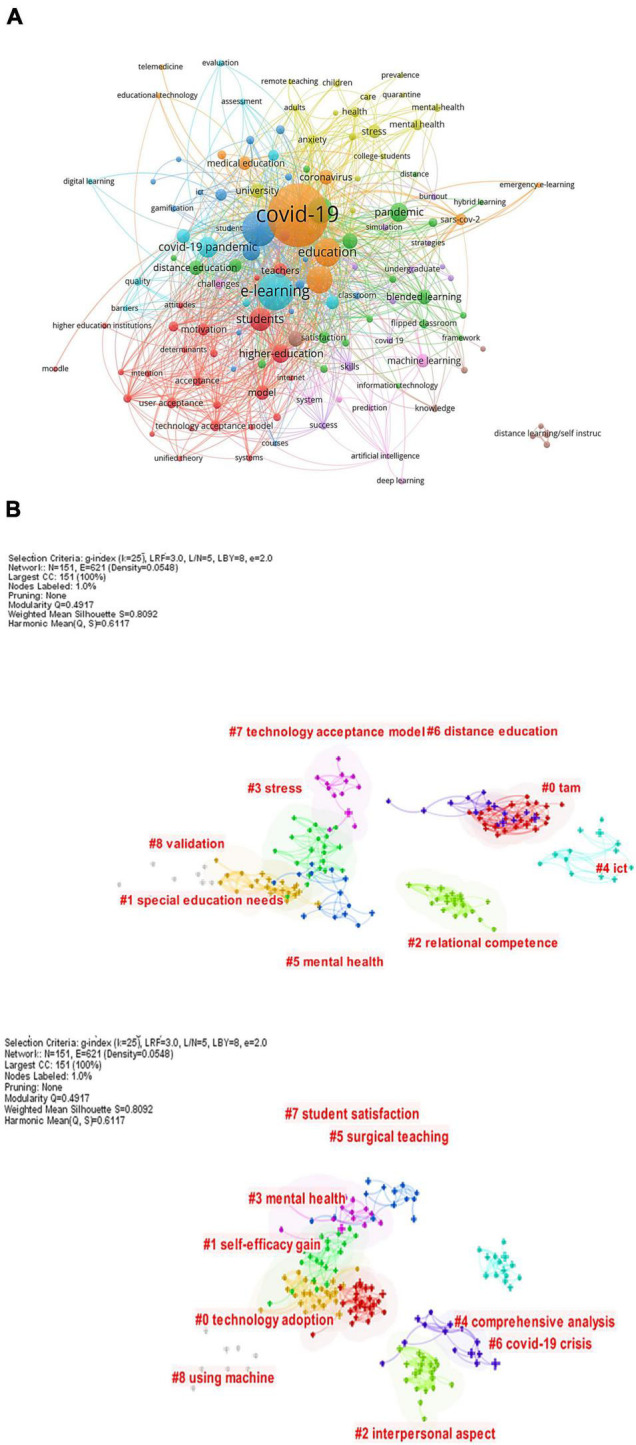
**(A)** Network of keywords (VOSviewer outputs). **(B)** Network of keywords (CiteSpace outputs).

Figures 2A,B and Supplementary Table 1 present the most frequent keywords that have been repeated more than five, which amounted to 131.

Figure 2A shows nine sub-areas (clusters) for research in e-learning within higher education during the era of COVID-19. First, the red cluster shows searches related to the following: higher education, students, motivation, attitudes, systems, technology acceptance model, and user acceptance. Second, the green cluster shows searches related to the pandemic, blended learning, online learning, hybrid learning, flipped classrooms, virtual learning, and distance education. Third, the navy-blue cluster shows searches related to higher education online, online teaching, online assessment, formative assessment. Fourth, the yellow cluster relates to stress, health, care, quarantine, mental health, anxiety, college students, adults, children. Fifth, the violet cluster shows searches related to surgery, surgical education, skills, strategies, student satisfaction, and simulation. Sixth, the light blue cluster shows searches related to e-learning, performance, quality, remote learning, digital learning, assessment, evaluation. Seventh, the orange cluster shows searches related to education, Covid-19, coronavirus, sars-cov-2, distance learning, medical education. Eighth, the brown cluster included: computer-based learning, self-instruction/distance learning, internet/web-based education, curriculum, knowledge, science, and technology. Finally, the pink clusters showed searches related to artificial intelligence, machine learning, and deep learning. The researcher can also take these subfields as topics for research in e-learning, especially the last cluster, which formed a recent research trend for many scholars (Bhardwaj et al., 2021; Kashive et al., 2021; Rasheed and Wahid, 2021).

Figure 2B shows that the research on this topic requires focusing on several issues. These are the most frequently mentioned keywords in Supplementary Table 1, including COVID-19 crisis, technology acceptance model (TAM), distance education, stress, ICT, special education needs, mental health, student satisfaction, surgical teaching, self-efficacy, technology adoption, using the machine, and e-learning. At the same time, many studies used different terms to express the same meaning, such as interactive learning, online learning, and Distance learning. This is similar to what was found in previous studies on e-learning (Chiang et al., 2010; Cheng et al., 2014; Bai et al., 2020; Fatimah et al., 2021).

### Reference Authors

**FIGURE 3:**
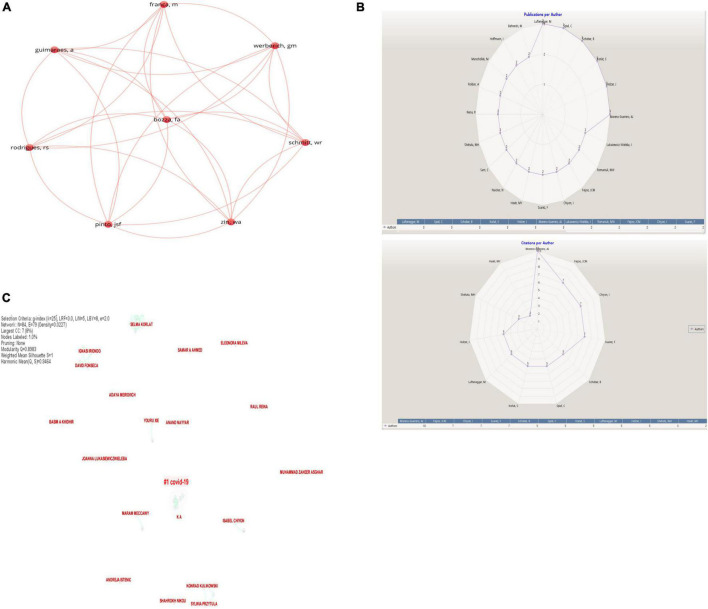
**(A)** Network of authors (VOSviewer outputs). **(B)** Publications and citations per author (KnowledgeMatrix Plus outputs). **(C)** Network of cited authors in COVID-19 (CiteSpace outputs).

Figures 3A–C show the network of the most referenced authors on the topic of “E-learning in higher education in COVID-19” based on co-authorship:

Figure 3A shows that there is a research partnership between eight authors. The co-authorship is the affiliation and the country: Fernando Augusto Bozza, Rosana Souza Rodrigues, Walter Araujo Zin, Alan Guimaraes and Gabriel Madeira Werberich, Federal University of Rio de Janeiro, Brazil. Joana Sofia F. Pinto, Willian Reboucas Schmitt and Manuela Franca, Complexo Hosp Univ Porto, Radiol Dept, Porto, Portugal. As for the rest, they have separate and individual publications. Figures 3A–C present the top authors based on publications and citations.

Figure 3B shows that the first author on this topic on “E-learning in higher education in COVID-19” is Antonio José Moreno-Guerrero, Univ Granada, Dept Didact & Sch Org, Spain. Among this research, we find “Impact of Educational Stage in the Application of Flipped Learning: A Contrasting Analysis with Traditional Teaching” (Pozo Sánchez et al., 2019). We also find research on e-learning in mathematics teaching: an educational experience in adult high school (Moreno-Guerrero et al., 2020) as well as research on the following: the effectiveness of innovating educational practices with flipped learning and remote sensing in earth and environmental sciences (López Núñez et al., 2020); machine learning and big data and their impact on literature; a bibliometric review with scientific mapping in WoS; and a flipped learning approach as an educational innovation in water literacy (López Belmonte et al., 2020; López Núñez et al., 2020). Moreno-Guerrero talked about e-learning and did not discuss the COVID-19 (Moreno-Guerrero et al., 2020); otherwise, Lüftenegger discussed e-learning and COVID-19 (Holzer et al., 2021; Korlat et al., 2021; Pelikan et al., 2021).

Figure 3C shows that the most important authors searched in COVID-19 and touched on e-learning are Maram Meccawy, Isabel Chiyon, and Anand Nayyar among others.

### Reference Organizations

**FIGURE 4:**
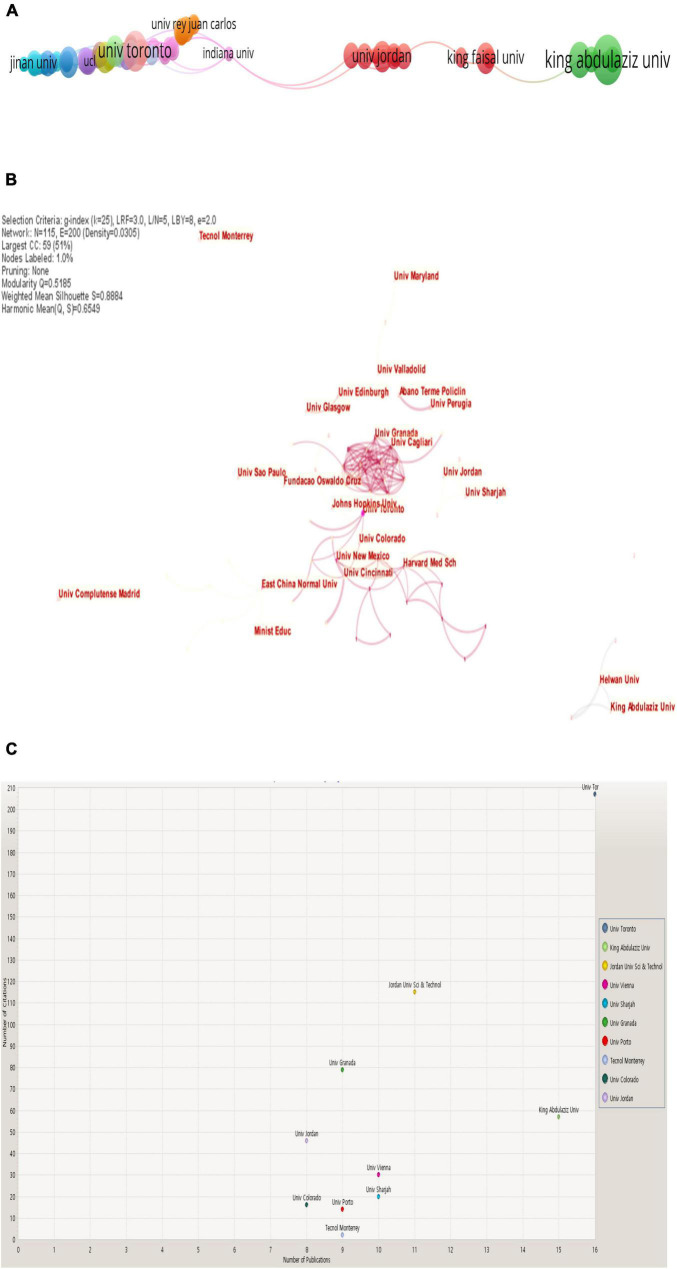
**(A)** Network of organizations (VOSviewer outputs). **(B)** Network of organizations (CiteSpace outputs).**(C)** Citations per publications by the organization (KnowledgeMatrix Plus outputs).

Figures 4A–C displays the most referenced organizations on the topic of “E-learning in higher education in COVID-19” based on publications, citations, and co-authorship.

Figures 4A–C demonstrate that the leading research organization for publications, citations, and co-authorship on this topic is the University of Toronto with 16 publications and 207 citations, followed by the University of King Abdulaziz with 15 publications and 57 citations the Jordan University of Science and Technology with 11 publications and 115 citations, then the University of Vienna with 10 publications and 30 citations, then the University of Sharjah with 10 publications and 20 citations, then the University of Granada with 9 publications and 79 citations, then the University of Porto with 9 publications and 14 citations, then Monterrey Institute of Technology and Graduate Studies with 9 publications and 2 citations, then the University of Jordan with 8 publications and 46 citations, and finally, the University of Colorado with 8 publications and 16 citations. That is due to several reasons, including the interest of these organizations in publishing in the WoS database. Then their interest in publishing in the subject of the study. We thus find it among the top 500 universities.^1^

### Reference Countries

**FIGURE 5:**
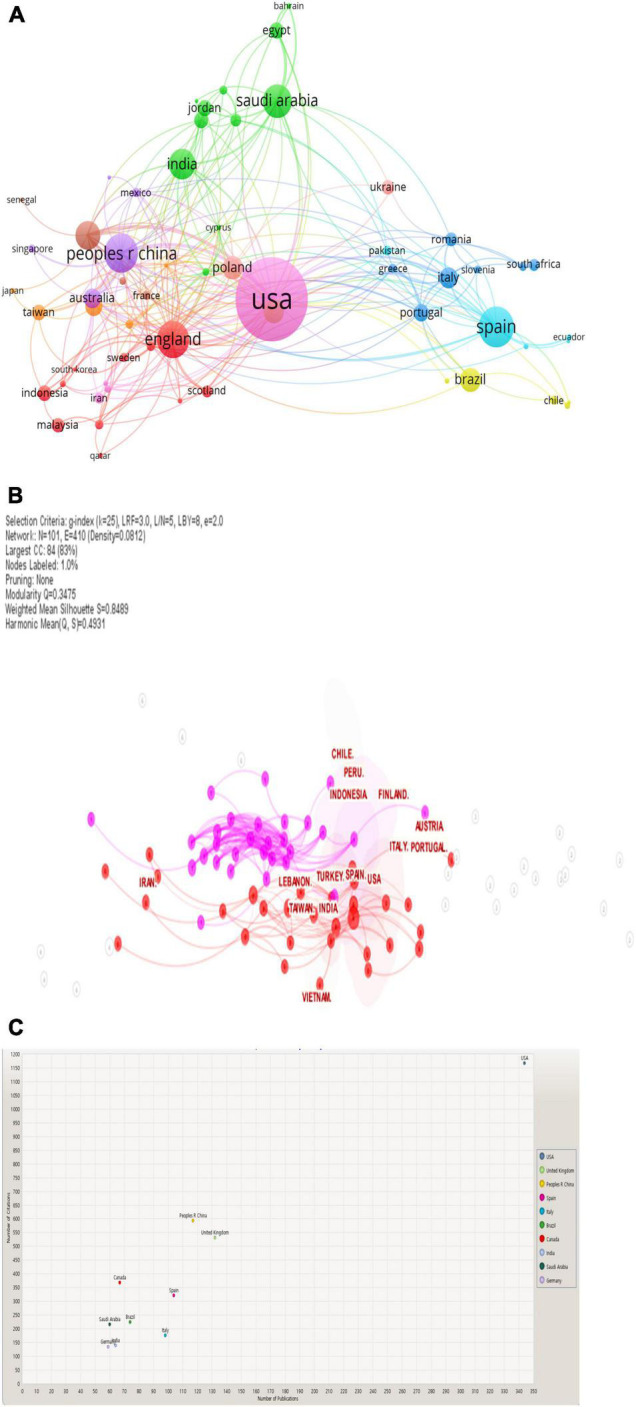
**(A)** Network of countries (VOSviewer outputs). **(B)** Network of countries (CiteSpace outputs). **(C)** Citations per publications by country (KnowledgeMatrix Plus outputs).

Figures 5A–C display the most referenced countries on the topic of “E-learning in higher education in COVID-19” based on publications, citations, and co-authorship.

Figures 5A–C illustrate that the top countries for publications, citations, and co-authorship in this topic are as follows: the United States with 344 publications and 1,167 citations, the United Kingdom with 132 publications and 530 citations, China with 117 publications and 592 citations, Spain with 104 publications and 321 citations, Italy with 98 publications and 175 citations, Brazil with 74 publications and 224 citations, Canada with 67 publications and 368 citations, India with 64 publications and 139 citations, Saudi Arabia with 60 publications and 216 citations, and Germany with 59 publications and 133 citations. These show extensive collaboration, especially between the United States and the United Kingdom with 11 collaborations, between the United States and Canada with 10 collaborations, and between the United States and China with 9 collaborations; other countries show an average of 3–5 collaborations.

### Discussion

The results of the bibliometric analysis showed that there are nine sub-fields of research within a topic: motivation and students’ attitudes to e-learning systems in higher education (technology acceptance model), comparison between blended learning and virtual learning, online assessment versus formative assessment of students in higher education, stress, anxiety, and mental health of college students in COVID-19, surgical education strategies to develop students’ skills, quality and performance of higher education strategies of e-learning in COVID-19, challenges of medical education and distance learning during COVID-19, and changing higher education curricula using technology.

Finally, using artificial intelligence, machine learning, and deep learning to transform the e-learning Industry, this final sub-field formed a recent research trend for many scholars (Bhardwaj et al., 2021; Kashive et al., 2021; Rasheed and Wahid, 2021).

The bibliometric study shows that the first author in e-learning is Antonio José Moreno-Guerrero, Univ Granada, Dept Didact & Sch Org, and Spain. His writings (Pozo Sánchez et al., 2019; López Núñez et al., 2020; Moreno-Guerrero et al., 2020) are considered a useful reference in e-learning and blended learning. Therefore, Marko Lüftenegger is one of the most influential author in the topic of “E-learning in higher education in COVID-19” (Holzer et al., 2021; Korlat et al., 2021; Pelikan et al., 2021)

The results of the bibliometric analysis showed that the top research organizations in this domain are as follows: the University of Toronto, the University of King Abdulaziz, Jordan University of Science and Technology, the University of Vienna, the University of Sharjah, the University of Granada, the University of Porto, Monterrey Institute of Technology and Graduate Studies, the University of Jordan, and the University of Colorado. The results also illustrate that the top countries are: United States, United Kingdom, China, Spain, Italy, Brazil, Canada, India, Saudi Arabia, Germany, due to several reasons, including the interest of these organizations and countries in publishing in the Web of Science database and their interest in publishing in the subject of the study.

Our research overlaps with that of López-Belmonte et al. (2021), who tried to investigate the development of e-learning in higher education in the academic literature listed on the WoS. The same analysis, as well as bibliometric analysis, was carried out. The findings revealed no set path for research because of the research on e-learning in higher education, recent creation, and a scarcity of relevant research. According to the results of the bibliometric analysis, the study was aimed at determining acceptance and implementation of the educational curriculum in the teaching and learning processes.

## Conclusion

This paper discusses the use of a bibliometric approach to track e-learning trends in higher education during the COVID-19 pandemic through the WoS database. From a methodological perspective, our proposed approach can visually represent the temporal links of the most cited articles internally in various streams and provide a comprehensive overview of the evolution of topics in the WoS database. Also, direct citation network analysis enables researchers to test articles important in e-learning and get a comprehensive overview of the issues published.

The study provided an insight into the world’s e-learning research in terms of mapping research publications. A scientific study was conducted using 602 e-learning documents from 2020 to 2021, and these were obtained through the WoS database. Over the years, the analysis identified trends in contributions in this area and headline sources for most researchers and leading institutions. The study is convergent with many previous studies in this area, including Chiang et al. (2010), Hung (2012), Cheng et al. (2014), Tibaná-Herrera et al. (2018b), Fatima and Abu (2019), Bai et al. (2020), and Mashroofa et al. (2020). However, our study relies on many software to compare various theoretical models and networks of e-learning.

Based on the analysis data’s inference, growth trends in research publishing in e-learning of different forms have increased in recent years, especially so for the last 2 years (230 in 2020 and 386 in 2021). The significant findings of the bibliometric analysis are as follows: there are nine sub-fields of study in the subject of “E-learning in higher education in COVID-19,” and the prominent authors in this area are as follows: Antonio José Moreno-Guerrero and Marko Lüftenegger; the University of Toronto Canada is the most frequently cited organization in this domain; the United States is the leading country in terms of publications and citations; and the sub-field of artificial intelligence, machine learning, and deep learning to transform the eLearning Industry has emerged as a recent research trend for many scholars.

The study examined a very important topic, which is one of the current topics, “e-learning in higher education during COVID-19,” using bibliometric analysis of 602 studies published in Web of Science databases from 2020 to 2021. We found that the study sample should be larger; it needs further studies and a longer time, especially when we analyze citation, and research on this topic will thus continue in future years. Also, there are many tools and methods used in the bibliometric analysis that were not used in our study, including what has been mentioned (Tibaná-Herrera et al., 2018b; Gul et al., 2020; López-Belmonte et al., 2021; Rashid et al., 2021).

The findings of this study will assist interested academics and educational policymakers (Brika et al., 2021) in the field of e-learning in understanding the current state of e-learning and identifying the different research trends in light of COVID-19. Additionally, it will serve as the beginning point for new research during the COVID-19 crisis, which will examine various problems and trends.

The findings of this research may help evaluate e-learning institutions’ quality and promote future educational trends. The findings may be utilized by e-learning institutions to evaluate quality as strategic dimensions and policy makers’ vision.

## Data Availability Statement

The original contributions presented in the study are included in the article/Supplementary Material, further inquiries can be directed to the corresponding author.

## Author Contributions

All authors contributed to the design and implementation of the research, performed the revision, verified the analytical methods, supervised the findings of this work, discussed the results, and contributed to the final manuscript.

## Conflict of Interest

The authors declare that the research was conducted in the absence of any commercial or financial relationships that could be construed as a potential conflict of interest.

## Publisher’s Note

All claims expressed in this article are solely those of the authors and do not necessarily represent those of their affiliated organizations, or those of the publisher, the editors and the reviewers. Any product that may be evaluated in this article, or claim that may be made by its manufacturer, is not guaranteed or endorsed by the publisher.

## Footnotes

1 https://www.topuniversities.com/university-rankings/world-university-rankings/2021, https://www.timeshighereducation.com/world-university-rankings/2021/world-ranking#!/page/0/length/25/sort_by/rank/sort_order/asc/cols/stats
