# Supplementary figures and images for: E-Learning Research Trends in Higher Education in Light of COVID-19: A Bibliometric Analysis
Source: Front Psychol. 2022 Mar 3;12:762819. doi: 10.3389/fpsyg.2021.762819 (PMC8929398; doi:10.3389/fpsyg.2021.762819)

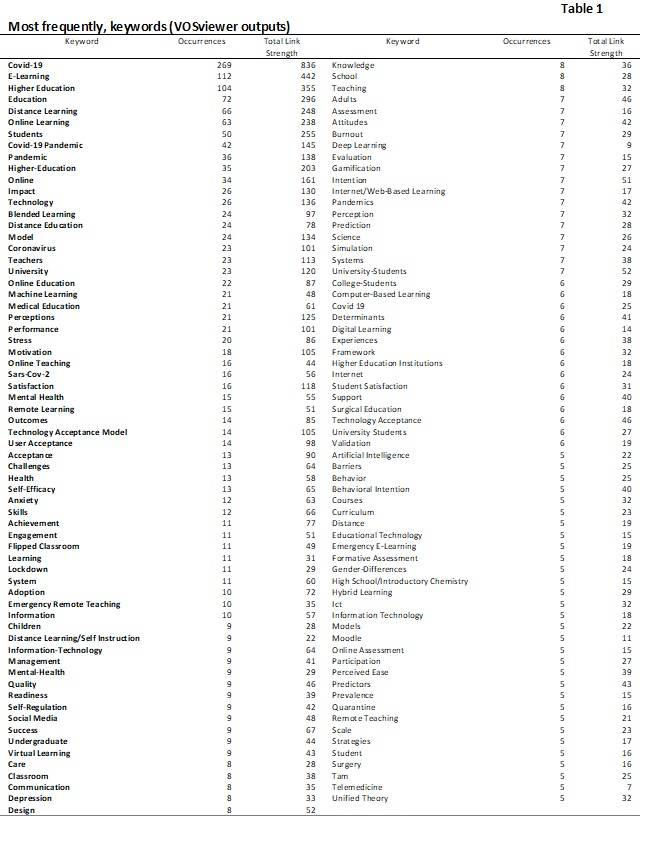

Supplement: Supplementary file 1 [file Image_1.JPEG]
